# Mechanism of Robo1 in the pentylenetetrazol‐kindled epilepsy mouse model

**DOI:** 10.1002/ibra.12127

**Published:** 2023-08-15

**Authors:** Zheng Liu, Wei Huang, Man‐Min Zhu, Zhong‐Xiang Xu, Zu‐Cai Xu, Chang‐Yin Yu, Hao Huang

**Affiliations:** ^1^ Department of Neurology Affiliated Hospital of Zunyi Medical University Zunyi China

**Keywords:** dendritic spine, epilepsy, hippocampus, Robo1

## Abstract

The neural network hypothesis is one of the important pathogenesis of drug‐resistant epilepsy. Axons guide molecules through synaptic remodeling and brain tissue remodeling, which may result in the formation of abnormal neural networks. Therefore, axon guidance plays a crucial role in disease progression. However, although Robo1 is one of the important components of axon guidance, the role of Robo1 in epilepsy remains unclear. In this study, we aimed to explore the mechanism of Robo1 in epilepsy. Male adult C57BL/6 mice were intraperitoneally injected with pentylenetetrazol to establish an epilepsy model. Lentivirus (LV) was given via intracranial injection 2 weeks before pentylenetetrazol injection. Different expressions of Robo1 between the control group, LV‐mediated Robo1 short hairpin RNA group, empty vector control LV group, and normal saline group were analyzed using Western blot, immunofluorescence staining, Golgi staining, and video monitoring. Robo1 was increased in the hippocampus in the pentylenetetrazol‐induced epilepsy mouse model; lentiviral Robo1 knockdown prolonged the latency of seizure and reduced the seizure grade in mice and resulted in a decrease in dendritic spine density, while the number of mature dendritic spines was maintained. We speculate that Robo1 has been implicated in the development and progression of epilepsy through its effects on dendritic spine morphology and density. Epileptic mice with Robo1 knockdown virus intervention had lower seizure grade and longer latency. Follow‐up findings suggest that Robo1 may modulate seizures by affecting dendritic spine density and morphology. Downregulation of Robo1 may negatively regulate epileptogenesis by decreasing the density of dendritic spines and maintaining a greater number of mature dendritic spines.

## Graphical abstract

Mechanism diagram of Robo1 involved in epileptic seizures (draw by Figdraw) (Slit‐Robo GTPase activating protein [SRGAP], Rho GTPase activating protein 39 [ARHGAP39]，p21‐activated kinase 1 [PAK1], Ras‐related C3 botulinum toxin substrate 1 [Rac1], Ras homologous family member A [RhoA], cell division cycle 42 [CDC42], myosin9B [MYO9B], guanosine diphosphate [GDP], guanosine triphosphate [GTP])

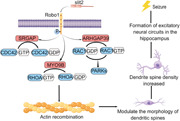

## 1 INTRODUCTION

Epilepsy is a common chronic neurological disorder. A typical characteristic of epilepsy is transient but highly synchronous abnormal discharges among neurons in the brain. There are currently about 70 million epilepsy patients worldwide.^1^ About 2.4 million new cases of epilepsy are confirmed each year.^2^ Studies have reported that epilepsy accounts for 0.7% of the global disease burden, bringing a serious economic burden to families and society.^3^, ^4^ Although more than 10 kinds of antiseizure drugs have good effects on controlling seizures worldwide, about 30% of patients still develop drug‐resistant epilepsy. Therefore, it is vital to understand the upstream mechanisms of epileptogenesis. This provides further evidence to explore the pathogenesis of epilepsy and new therapeutic targets.

Robo1 is a conserved transmembrane protein associated with development. The Robo family has four members that mediate a variety of neuronal responses, including neurogenesis, migration, branching, dendritic pattern formation, synaptogenesis, and axon guidance,^5^ which play an important role in angiogenesis and tumorigenesis^6^ and are involved in a variety of neurological diseases such as ischemic cerebrovascular disease^7^ and peripheral neuropathy.^8^ Robo1, a member of the Robo family, is widely expressed in the central nervous system.^9^ Slit is a member of the Slit family of neural guidance factors (Slit1–3) and Slit/Robo signaling is first identified in the nervous system.^10^

Most scientists believe that synaptic remodeling, mossy‐fiber sprouting, and morphological changes in dendritic spines constitute the main structural basis for excitatory neural circuits that produce abnormal neuronal discharge and recurrent seizures leading to epilepsy.^11^, ^12^ The Slit/Robo1 signaling pathway can control neural tube development by regulating the expression of genes associated with cell proliferation and differentiation.^13^ The Slit2/Robo1 signaling pathway regulates small GTPases of the Rho family (Rho GTPases) such as RhoA, Cdc42, and Rac, while Robo1/Slit2 regulates the reorganization of the actin cytoskeleton by modulating the activity of these proteins.^9^, ^14^ The actin network not only regulates the structural shape of neurons but also plays an important role in pre‐ and postsynaptic synaptic function,^15^ and these changes are associated with the onset of epilepsy. In a lithium‐pyrrolizidinium chloride‐induced rat model of epilepsy, Robo3 expression in the hippocampus is found to decrease over time during acute and chronic epilepsy in epileptic rats. The expression reached its lowest level during the chronic phase, suggesting that Robo3 may be involved in temporal lobe epilepsy.^16^

Based on the above study, since the role of Robo1 in epilepsy has not been investigated or fully illustrated, we used a variety of experimental methods and observed alterations in epileptic behavior in various groups of mice to explore the impact of activation of the Robo1 on dendritic spine morphology and density. Eventually, a new target and theoretical background are provided for the diagnosis and treatment of epilepsy.

## 2 MATERIALS AND METHODS

### 2.1 Animals

Six‐week‐old male mice (C57BL/6; Hunan Sleckingda Laboratory Animal Co., Ltd.) weighing 21–22 g were housed in an environment with a 12‐h light/dark cycle at 22°C. All procedures were approved by the Commission of Zunyi Medical University for Ethics of Experiments on Animals and implemented in accordance with international standards. The ethics review number is KLLYA‐2020‐097 (acquisition time: 2020.6.12).

### 2.2 Pentylenetetrazol (PTZ) modeling and intervention

Endogenous Robo1 expression in hippocampal neurons was diminished using lentivirus (LV)‐Robo1‐short hairpin RNA (shRNA). Three Robo1 LV (LV‐Robo1‐shRNA) sequences (pHS‐ACR0207: 5′‐CAGGAAGAGGGCCTATTTCCC‐3′ virus titer: 1.27e + 08 TU/mL; pHS‐ACR0208: 5′‐CAGGAAGAGGGCCTATTTCCC‐3′ virus titer: 1.03e + 08 TU/mL; pHS‐ACR0209: 5′‐CAGGAAGAGGCCTATTTCCC‐3′ virus titer: 1.16e + 08 TU/mL) and control LV (pHSBCRLW010 virus titer: 1.23e + 08 TU/mL) were designed by Hopson Biotechnology Co., Ltd. The LV‐Robo1‐shRNA gene sequence that demonstrated the highest expression of Robo1 in the hippocampus of mice was selected through Western blot analysis. Mice were anesthetized with sodium pentobarbital (50 mg/kg) and mounted onto the stereotaxic apparatus. The location of the hippocampal CA1 subfield (CA1) was determined by locating the bregma in transverse and longitudinal coordinates with reference to the stereotaxic atlas of the mice's brain.^17^ The injection site was located in the CA1 region of the hippocampus bilaterally. Specific indicators are listed below: 2.5 mm posterior to the bregma, 2.0 mm to the left and right of the median suture, and 1.20 mm in depth. LV of 2 μL was injected at a rate of 0.2 μL/min per measurement, controlled by an ultramicroinjection pump. After the injection, the syringe was maintained in situ for an additional 10 min to minimize reflux. The scalp was sutured and the mice were kept at body temperature until they woke up.

The PTZ model was established 2 weeks after LV injection. By continuous intraperitoneal injection of PTZ (35 mg/kg) for 28 days, a rat model of chronic epilepsy was established. Nonconvulsive status epilepticus was terminated using pentobarbital. In the following month, the mice were placed under a video monitoring system to record their seizure.

According to different experimental requirements, there were nine mice in each group, and 99 mice were required. The experimental groups are described as follows (Table 1).

**Table 1:** Experimental grouping and methods.

Group	Abbreviation	Methods
Normal control group	Control group	Laboratory mice did not receive any treatment.
Normal saline group	NS group	Normal saline was injected into the bilateral hippocampal CA1 region.
Empty vector control lentivirus group	LV‐GFP group	Empty virus was injected into the bilateral hippocampal CA1 region.
Lentivirus‐mediated Robo1 shRNA group	LV‐Robo1‐shRNA group	Lentivirus interference was injected into the bilateral hippocampal CA1 region.
Pentylenetetrazol (PTZ)‐kindled epilepsy mouse model group	Model group	A PTZ epilepsy model was established.
Pentylenetetrazol and empty vector control lentivirus	PTZ + LV‐GFP group	Empty virus was injected into the bilateral hippocampal CA1 region and then was kindled using PTZ.
Pentylenetetrazol and lentivirus‐mediated Robo1 shRNA group	PTZ + LV‐Robo1‐shRNA group	Lentivirus interference was injected into the bilateral hippocampal CA1 region and then was kindled using PTZ.

*Note*: The group column is the full name of the different experimental groups, the abbreviation column is the abbreviation of the corresponding group, and the methods column is the intervention method of the corresponding experimental group.

Abbreviations: GFP, green fluorescent protein; LV, lentivirus; shRNA, short hairpin RNA.

### 2.3 Behavioral observation of mice

Within 1 h after PTZ injection according to Racine grading criteria, scores were recorded each day for convulsive seizure. The seizure grade was recorded according to Racine's scales as follows: Stage 0, no response or behavior arrest; Stage 1, chewing or facial twitches; Stage 2, chewing and nodding or wet‐dog‐shaking; Stage 3, unilateral forelimb clonus; Stage 4, bilateral forelimb clonus and rearing; and Stage 5, bilateral forelimb clonus, rearing, and falling.^18^

### 2.4 Immunofluorescence staining

Immunofluorescence was performed as previously described by our laboratory.^19^ Briefly, brain tissue sections were fixed in 4% polyformaldehyde for 1 min. The sections were then washed three times with phosphate‐buffered saline (PBS). Next, the sections were permeated with 0.4% Triton X‐100 for 30 min and blocked with goat serum working liquid (Shanghai Biyuntian Biotechnology Co., Ltd.) for 1 h. Afterward, the sections were incubated overnight with mixed primary antibodies at 4°C. After that, they were washed in PBS to remove unbound primary antibodies and incubated with secondary antibodies in darkness at room temperature for 1 h. The primary antibodies included rabbit anti‐Robo1 (1:100; Abcam) and mouse anti‐NeuN (1:200; Wuhan Boster Biological Technology). The secondary antibodies used for fluorophore conjugation were goat anti‐mouse Alexa Fluor 596 (1:100; Abcam) and goat anti‐rabbit Alexa Fluor 488 (1:100; Wuhan Boster Biological Technology). 4′,6‐Diamidino‐2‐phenylindole (DAPI) solution was then added to the slices and incubated for 5 min at room temperature. Images were captured by confocal laser scanning microscopy (Leica). The fluorescence intensity was analyzed using ImagePro Plus 6.0 (Media Cybernetics). Colocalization analyses were performed using a Zeiss confocal microscope.

### 2.5 Western blot analysis

Hippocampal tissues samples were collected for Western blot analysis. Protein samples were separated using 10% sodium dodecyl sulfate–polyacrylamide gel electrophoresis gels and electrotransferred onto 0.22 μm polyvinylidene fluoride membranes. The membranes were blocked with 5% nonfat dry milk in Tris‐buffered saline with Tween (TBST) at room temperature for 2 h. Then, the samples were incubated with a primary antibody (Robo1: 1:1000, β‐tubulin: 1:8000) overnight at 4°C. The blots were washed three times and placed with secondary antibodies (dilution ratio: 1:8000) from the same species at room temperature for 2 h. The bands were then washed in TBST. Then, an enhanced chemiluminescence system (Advansta) was applied to visualize the bands and the ChemiDoc Touch Imaging System for imaging (Bio‐Rad). The gray scale values were analyzed using Image J (edition: 1.54d). Using β‐tubulin gray scale values as an internal reference, the relative expression of target proteins was calculated.

### 2.6 Golgi staining

Golgi staining was performed using the protocol recommended by the FD Rapid Golgistain Kit (FD Neuro Technologies Inc.). Briefly, the mice brains were immersed in a mixture of Solutions A and B at room temperature and protected from light for 14 days after dissection. Subsequently, the brains were transferred to Solution C and soaked for 7 days. Slices (100‐μm thick) were prepared and stained according to the manufacturer's instructions. Image acquisition was carried out by orthographic microscopy, and the number of dendritic spines was calculated by Image J (edition: 1.54d).

### 2.7 Statistical analysis

The study was conducted by researchers following the principle of randomization and blinding. Experiments were performed at least in triplicate (for each biological replicate, *n* ≥ 3). All data were analyzed using the GraphPad Prism (version 6.01) and SPSS (version 18.0). Student's *t*‐test was used for comparisons between the two groups. One‐way analysis of variance (ANOVA) was used for multiple groups followed by Tukey's test, and *p* < 0.05 was statistically significant. In graphs, statistical significance is indicated by an asterisk.

## 3 RESULT

### 3.1 Robo1 expression and distribution in hippocampal tissues of normal and epileptic mice models

**Figure 1:**
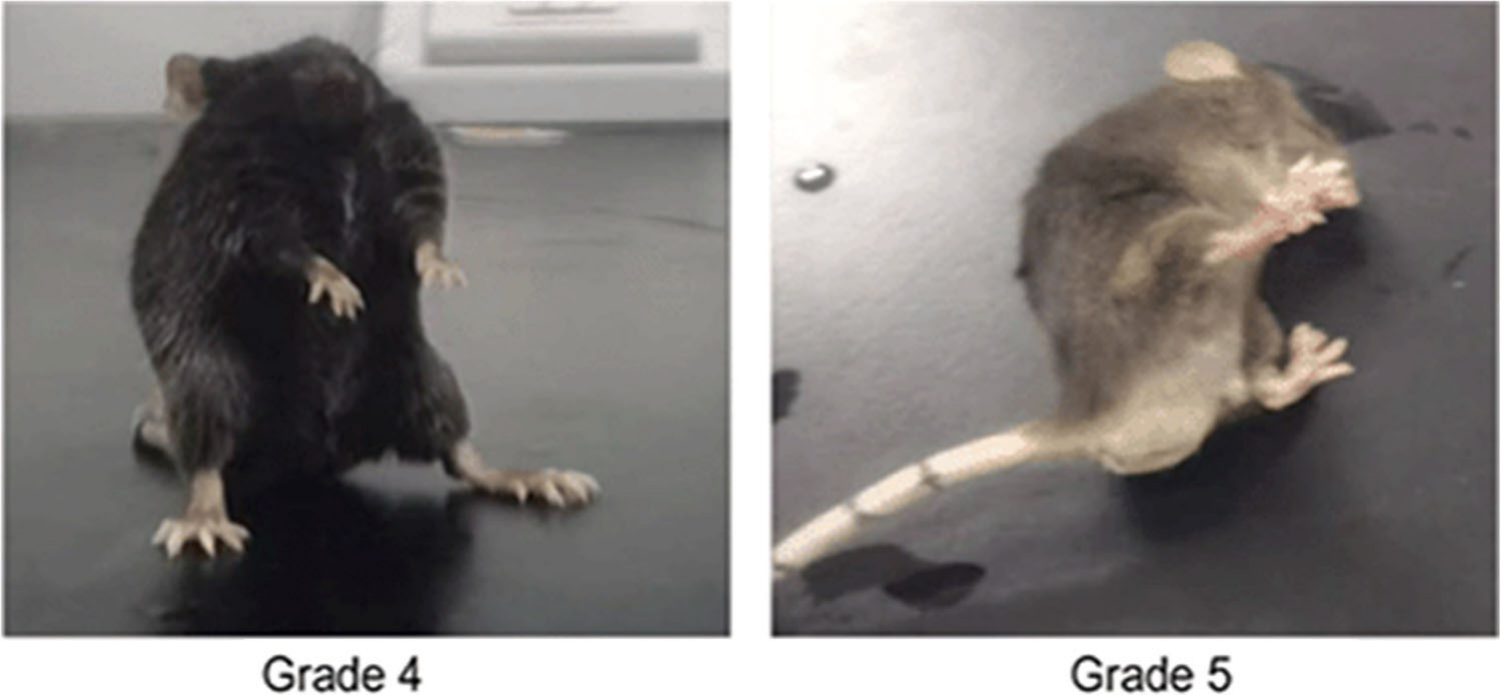
Images of the modified Racine attack score in mice. (Grade 4: represented by bilateral forelimb clonus and rearing; Grade 5: falling is accompanied by paroxysmal tonic‐clonic limbs). [Color figure can be viewed at wileyonlinelibrary.com]

**Figure 2:**
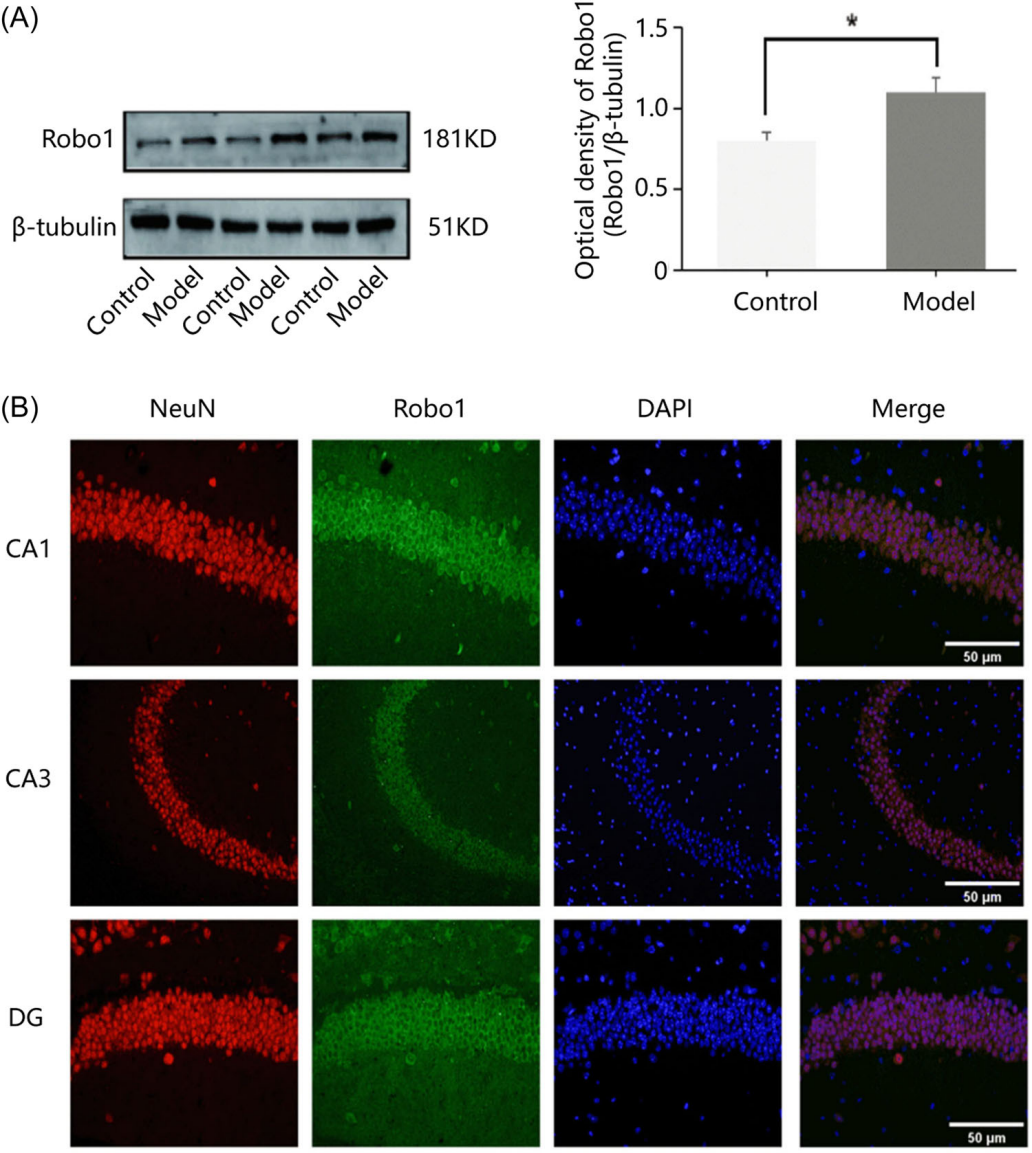
Robo1 expression and distribution in epilepsy. (A) Expression of Robo1 in the control group, model group, and the ratio of Robo1/β‐tubulin statistical results (*Note*: *Compared with the control group *p* < 0.05, Student's *t*‐test). (B) Immunofluorescence labeling was used to examine the localization of Robo1 in all three regions (CA1, CA3, DG) of the hippocampus in epileptic mice (×400). CA1, hippocampal CA1 subfield; CA3, hippocampal CA3 subfield; DG, dentate gyrus; KD, Kilodalton; DAPI, 4′,6‐diamidino‐2‐phenylindole. [Color figure can be viewed at wileyonlinelibrary.com]

Robo1, as described previously, was expressed in the nervous system. However, whether its expression and distribution in hippocampal regions are closely associated with epilepsy remains unknown. First, we modeled PTZ epilepsy. Racine Grade 4–5 was defined as successful modeling, and mice with Grade 4–5 were selected for testing (Figure 1). We first examined the expression levels of Robo1 by Western blot analysis in epileptic models. The intensity ratio of Western blot analysis between Robo1 and β‐tubulin in each group was 0.83 ± 0.78 and 1.13 ± 0.78, respectively. Robo1 was found to be basally expressed in the normal group and differentially expressed in the model group. The expression was significantly higher in the model group than that of the normal group (*p* < 0.05) (Figure 2A). To further precisely localize Robo1 in epilepsy, we examined the cellular localization of Robo1 in epileptic brain tissues by immunofluorescence double antibody labeling. Robo1 was colocalized with neuronal nuclei (NeuN; a marker of neurons), which does not overlap with the cell nuclear standard DAPI. Robo1 is mainly expressed in the cytosol (Figure 2B).

Taken together, Robo1 expression is increased in the epileptic brain and colocalizes with neurons in the epilepsy‐related hippocampal regions, suggesting that Robo1 may be involved in epileptic activity.

### 3.2 Expression of Robo1 in the hippocampus of mice after LV intervention

**Figure 3:**
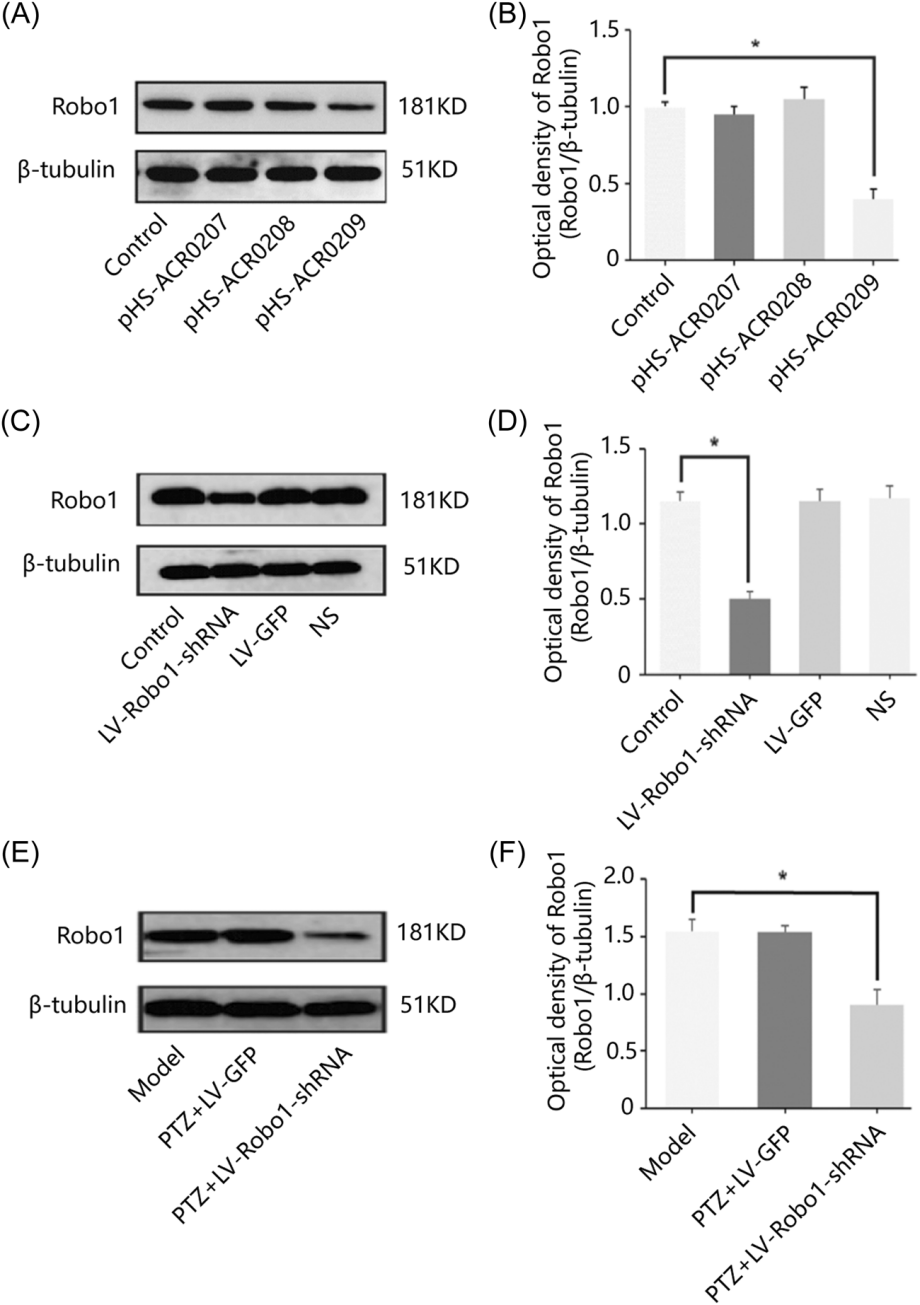
Protein levels of Robo1 in mice hippocampus. (A) Expression of Robo1 in the control group, pHS‐ACR0207 group, pHS‐ACR0208 group, and pHS‐ACR0209 group. (B) Quantification of Robo1 expression in the control group, pHS‐ACR0207 group, pHSACR0208 group, and pHS‐ACR0209 group (*n* = 5 in each group, *p < 0.05, one‐way ANOVA). (C) Expression of Robo1 in the control group, LV‐Robo1‐shRNA group, LV‐GFP group, and NS group. (D) Quantification of Robo1 expression in the mouse hippocampus after lentiviral intervention (*n* = 9 in each group; *p < 0.05, one‐way ANOVA). (E) Expression of Robo1 in the model group, PTZ + LV‐Robo1‐shRNA group, and PTZ + LV‐GFP group. (F) Quantification of Robo1 expression in the mouse hippocampus in the model group, PTZ + LV‐Robo1‐shRNA group, and PTZ + LV‐GFP group (*n* = 9 in each group; *p < 0.05, one‐way ANOVA). LV, lentivirus; PTZ, pentylenetetrazol; shRNA, short hairpin RNA.

Changes in the expression of Robo1 in epilepsy could be either a phenomenon caused after epileptogenesis or a cause of seizures. First, the effect of the three LV‐Robo1‐shRNA sequences (pHS‐ACR0207; pHS‐ACR0208; pHS‐ACR0209) on the expression of Robo1 in mice CA1 was examined with Western blot analysis. The transfection efficiency showed the expression of the ratio of Robo1 and β‐tubulin immunoblotting strength in the control group: 0.96 ± 0.07; pHS‐ACR0207 group: 0.84 ± 0.12; pHS‐ACR0208 group: 0.99 ± 0.25; and pHS‐ACR0209 group: 0.42 ± 0.13 (Table 2). The effect of three LV‐Robo1‐shRNA sequences on Robo1 expression in mouse CA1 showed that the pHS‐ACR0209 sequence was significantly lower than pHS‐ACR0207 and pHS‐ACR0208, so the pHS‐ACR0209 sequence was used for subsequent experiments (Figure 3A,B).

**Table 2:** Effect of the three LV‐Robo1‐shRNA sequences on the expression of Robo1 in mice CA1 (mean ± standard deviation).

Group	Ratio of gray (Robo1/β‐tubulin)
Control group	0.96 ± 0.07
pHS‐ACR0207 group	0.84 ± 0.12
pHS‐ACR0208 group	0.99 ± 0.25
pHS‐ACR0209 group	0.42 ± 0.13^a^

Abbreviations: LV, lentivirus; shRNA, short hairpin RNA.

^a^ Compared with the control group *p* < 0.05.

To control a single variable, the experimental groups were controlled with LV, empty virus, and normal saline. Afterward, the mice were randomly divided into four groups with nine mice in each group. Therefore, after 1 week of bilateral hippocampal stereotactic injection of LV, the expression of Robo1 in the control group, LV‐Robo1‐shRNA group, LV‐GFP group, and NS group was observed. The Robo1 to β‐tubulin immunoblot intensity ratios were 1.15 ± 0.18, 0.56 ± 0.14, 1.11 ± 0.25, and 1.16 ± 0.28 in each group (Table 3). Robo1 expression was significantly lowered in the LV‐Robo1‐shRNA group than in the other three groups, where the difference between the LV‐Robo1‐shRNA group and control group was statistically significant (*p* < 0.05) (Figure 3C,D). The PTZ model was constructed after 2 weeks of intervention with LV‐mediated Robo1‐shRNA in bilateral hippocampal CA1 regions. We observed the expression of Robo1 in the hippocampus of mice in the model, PTZ + LV‐GFP, and PTZ + LV‐Robo1‐shRNA groups. The immunoblotting strength ratios of Robo1 and β‐tubulin in each group were 1.57 ± 0.29, 1.55 ± 0.15, and 0.86 ± 0.35, respectively (Table 4). The PTZ + LV‐Robo1‐shRNA group expressed significantly less than the model and PTZ + LV‐GFP groups, and the difference between the PTZ + LV‐Robo1‐shRNA group and model group was significant (*p* < 0.05) (Figure 3E,F). It can be seen that Robo1 may affect epileptic seizures.

**Table 3:** Expression of Robo1 after 1 week of bilateral hippocampal stereotactic injection of lentivirus in each group (mean ± standard deviation).

Group	Ratio of gray (Robo1/β‐tubulin)
Control group	1.15 ± 0.18
LV‐Robo1‐shRNA group	0.56 ± 0.14^a^
LV‐GFP group	1.11 ± 0.25
NS group	1.16 ± 0.28

Abbreviations: GFP, green fluorescent protein; LV, lentivirus; NS, normal saline; shRNA, short hairpin RNA.

^a^ Compared with the control group *p* < 0.05.

**Table 4:** Expression of Robo1 after 2 weeks of bilateral hippocampal stereotactic injection of lentivirus in each group (mean ± standard deviation).

Group	Ratio of gray (Robo1/β‐tubulin)
Model group	1.57 ± 0.29
PTZ + LV‐GFP group	1.55 ± 0.15
PTZ + LV‐Robo1‐shRNA group	0.86 ± 0.35^a^

Abbreviations: GFP, green fluorescent protein; LV, lentivirus; PTZ, pentylenetetrazol; shRNA, short hairpin RNA.

^a^ Compared with the model group *p* < 0.05.

### 3.3 Effect of Robo1 on seizure latency and seizure class in epileptic mice

**Figure 4:**
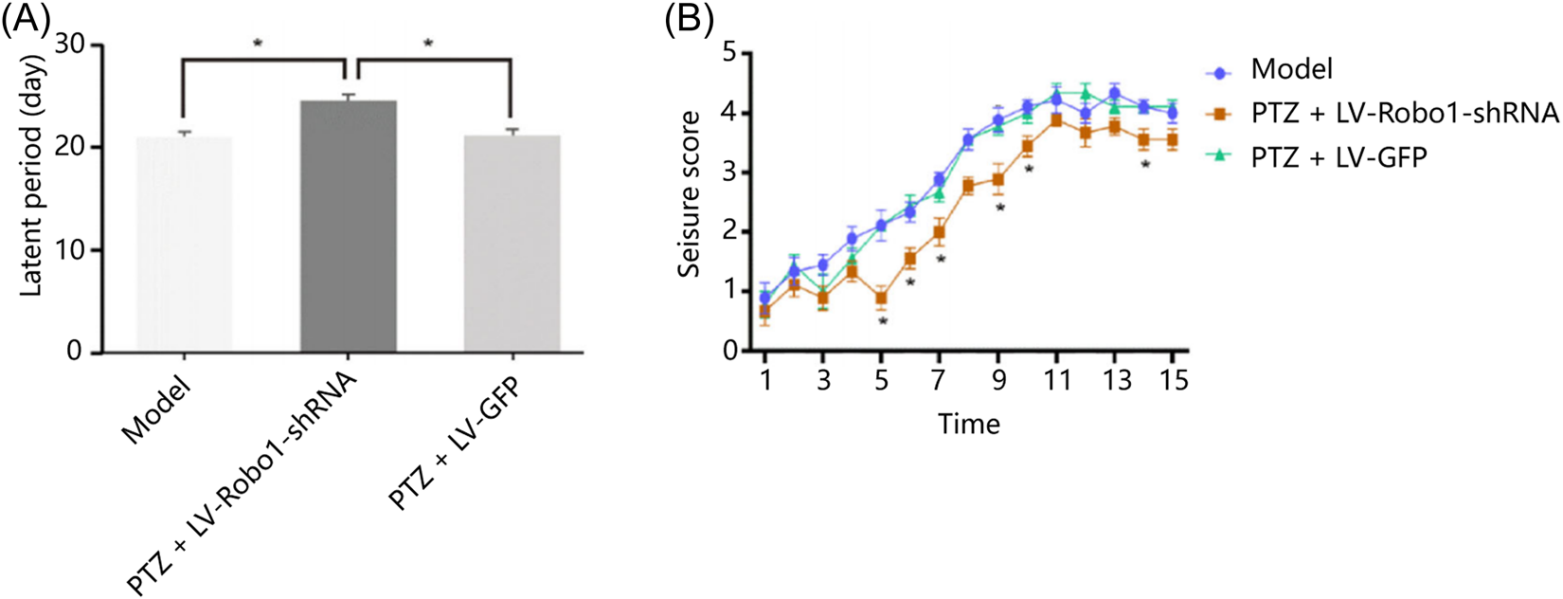
The influence of Robo1 interference on the seizure latency and class of mice. (A) Effect of Robo1 on the latent period of an epileptic seizure. (B) Robo1 influence the seizure score. *n* = 9 in each group; *p < 0.05, one‐way ANOVA. GFP, green fluorescence protein; LV, lentivirus; PTZ, pentylenetetrazol; shRNA, short hairpin RNA. [Color figure can be viewed at wileyonlinelibrary.com]

The behavioral results showed a latency of 21.33 ± 1.73 days for the model group, 25.11 ± 1.76 days for the PTZ + LV‐Robo1‐shRNA group, and 22.22 ± 2.10 days for the PTZ + LV‐GFP group (Table 5). A statistically significant difference was available in the PTZ + LV‐Robo1‐shRNA group with prolonged latency (Figure 4A, *p* < 0.05). The PTZ + LV‐Robo1‐shRNA group showed a significant decrease in the severity of seizure at 5–10 and 14 days after PTZ injection (Figure 4B). The latency time of the PTZ + LV‐Robo1‐shRNA group was significantly longer than that of the model group and the PTZ + LV‐GFP group (*p* < 0.05) (Figure 4A). This behavioral experiment suggests that the knockdown of Robo1 not only prolonged seizure latency but also reduced seizure class in mice (Figure 4). Therefore, the knockdown of Robo1 could prolong the incubation period of seizure and reduce the seizure grade.

**Table 5:** Latent period in the model group, PTZ + LV‐Robo1‐shRNA group, and the PTZ + LV‐GFP group (mean ± standard deviation).

Group	Latent period (day)
Model group	21.33 ± 1.73
PTZ + LV‐Robo1‐shRNA group	25.11 ± 1.76^a^
PTZ + LV‐GFP group	22.22 ± 2.10

Abbreviations: GFP, green fluorescent protein; LV, lentivirus; PTZ, pentylenetetrazol; shRNA, short hairpin RNA.

^a^ Compared with the model group and PTZ + LV‐GFP group *p* < 0.05.

### 3.4 Morphology and density of dendritic spines in the hippocampal CA1 region were detected by Golgi staining

**Figure 5:**
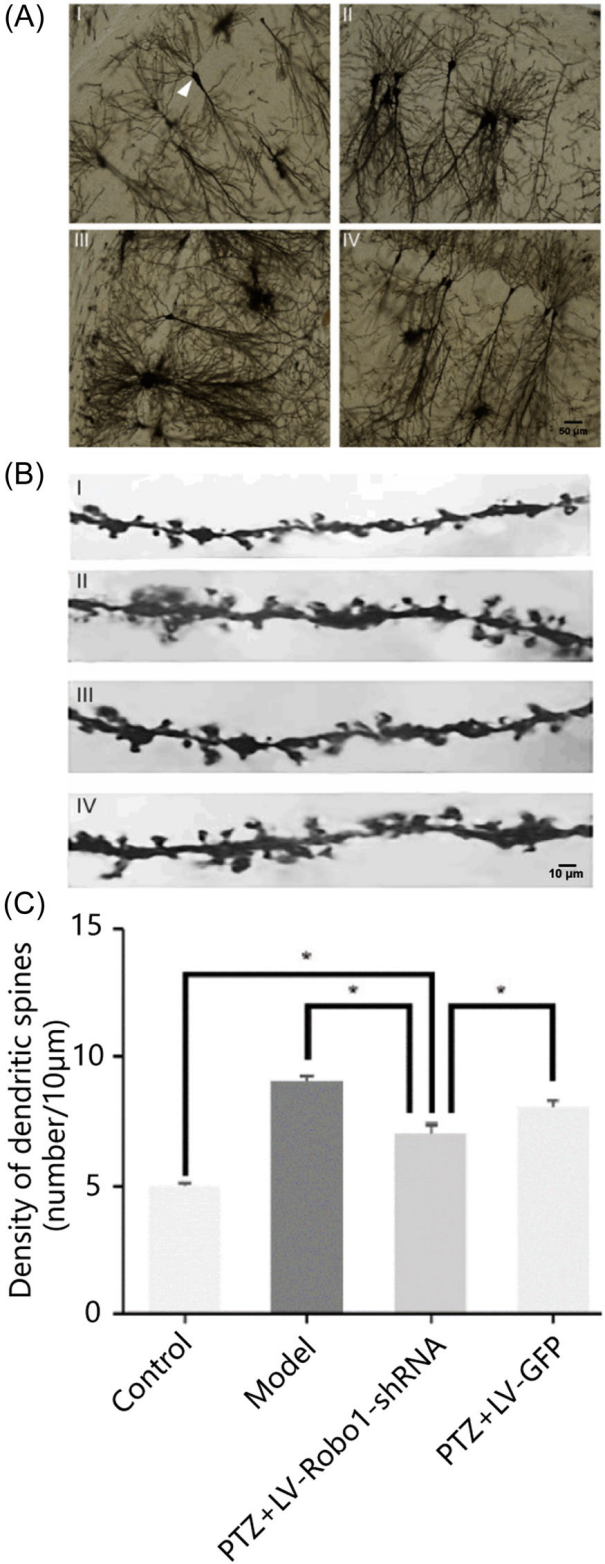
The density of dendritic spines in hippocampal CA1 region of each group were detected by Golgi staining. (A, B) Density of dendritic spines in the hippocampal CA1 region of each group was detected by Golgi staining (I: control group, II: model group, III: PTZ + LV‐Robo1‐shRNA group, and IV: PTZ + LV‐GFP group). Scale bar = 50 μm for A, 10 μm for B. (C)  Density of dendritic spines among different groups. *n* = 9; *p < 0.05, one‐way ANOVA. GFP, green fluorescent protein; LV, lentivirus; PTZ, pentylenetetrazol; shRNA, short hairpin RNA. [Color figure can be viewed at wileyonlinelibrary.com]

**Figure 6:**
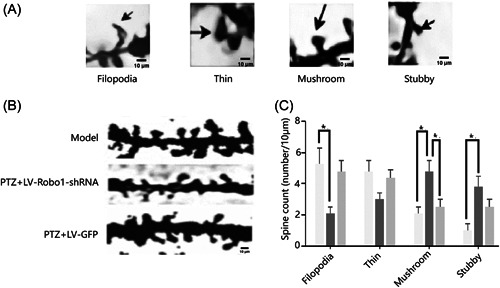
The morphology of dendritic spines in hippocampal CA1 neurons. (A) Different shapes of dendritic spines. (B, C) Morphology of dendritic spines in hippocampal CA1 neurons of each group was examined by Golgi staining. (Scale = 10 μm). *n* = 9; *p < 0.05, one‐way ANOVA. GFP, green fluorescent protein; LV, lentivirus; PTZ, pentylenetetrazol; shRNA, short hairpin RNA.

The morphology and density of dendritic spines in CA1 regions were observed under an optical microscope using Golgi staining after decapitating. The cytosol and apical dendrites were clearly visible (indicated by the arrow in Figure 5A). The length and number of dendritic spines in the hippocampal neurons from four groups were observed and their density and morphology were calculated by Image J (V1.8.0.112). The length and number of dendritic spines were calculated within 150–200 μm from the cytosol on the parietal dendritic trunk (Figure 5B). The results showed that the density of dendritic spines in each group was 4.84 ± 0.43, 9.50 ± 0.62, 6.72 ± 0.84, and 9.01 ± 0.78, respectively (Table 6). The PTZ + LV‐Robo1‐shRNA group was significantly lower than the model group and PTZ + LV‐GFP group (*p* < 0.05). By comparing PTZ + LV‐Robo1‐shRNA and PTZ + LV‐GFP groups compared to the control group, the dendritic spine density of hippocampal neurons was significantly higher in the model, and the differences were statistically significant (*p* < 0.05) (Figure 5C). The dendritic spine morphology was divided into filopodia, thin, mushroom, and stubby types (Figure 6A). The first two types were immature and the rest were mature. The results showed that the PTZ + LV‐Robo1‐shRNA group had significantly fewer filamentous pseudopod types and significantly shorter coarse types than those in the model group. The mushroom type was significantly more frequent in both the model group and the PTZ + LV‐GFP group and was statistically different (*p* < 0.05), and the stubby type was also significantly higher in the PTZ + LV‐Robo1‐shRNA group than in the model group, indicating that Robo1 could maintain more mature dendritic spines after knockout (Figure 6B,C).

**Table 6:** Density of dendritic spines in each group (mean ± standard deviation).

Group	Density of dendritic spines (per/μm)
Model group	9.50 ± 0.62
PTZ + LV‐Robo1‐shRNA group	6.72 ± 0.84^a^
PTZ + LV‐GFP group	9.01 ± 0.78
Control group	4.84 ± 0.43

Abbreviations: GFP, green fluorescent protein; LV, lentivirus; PTZ, pentylenetetrazol; shRNA, short hairpin RNA.

^a^ Compared with the model group and PTZ + LV‐GFP group *p* < 0.05.

## 4 DISCUSSION

In the present study, we first find that Robo1 may be involved in the disease process in PTZ‐ignited mice. Then, we report a novel finding that the knockout of Robo1 by LV reduces seizure latency and seizure grade, so the knockout affects the morphology and density of dendritic spines and contributes to the development of epilepsy.

An epileptic seizure is defined as a sudden occurrence of transient signs and symptoms caused by abnormal and excessive or synchronous neuronal activities in the brain.^20^ Its etiology and pathogenesis are complex, involving ion channels, synapse‐associated proteins, genes, and other factors.^21^, ^22^, ^23^ The most important pathological changes are synaptic remodeling of neurons and the formation of afferent excitatory loops in the hippocampus. Dendritic spines are an important component in the formation of synapses between neurons. It is the main site that provides excitatory inputs to neurons. There are four forms, filopodia, thin, mushroom, and stubby. The first two are immature and the rest are mature. They are highly centralized postsynaptic structures containing a number of transmembrane neurotransmitter receptors and proteins with signaling and scaffolding functions. After the intervention of some proteins or genes, the mature dendritic spines and immature dendritic spines can be transformed into each other, and the density of dendritic spines can be changed. This is a manifestation of synaptic plasticity.^24^, ^25^, ^26^ Recurrent episodes of excessive neural activity can lead to changes in brain plasticity, including axon germination, synaptic recombination, and nerves. Changes in brain plasticity may lead to abnormal neural networks.^27^

The Robo (Roundabout) protein family is a family of four neural cell adhesion molecules. Experts on neural development are relatively concerned about Robo1, Robo2, and Robo3. Robo1 serves as the receptor of neuroguidance factor Slit2. The Slit2/Robo1 signaling pathway participates in the growth, migration, and distribution of axons of neurons in the central nervous system.^28^ In experiments using a human‐derived cell line, it was demonstrated to promote the interaction between the intracellular CC3 domain of Robo1 and Slit/Robo GTPase‐activating protein (srGAP1) via binding Slit to Robo, resulting in the inactivation of Cdc42. Cdc42 inactivation suppresses activation of the actin‐related protein 2/3 complex and neuronal Wiskott‐Aldrich syndrome protein (actin polymerization regulatory protein, N‐WASP), resulting in actin depolymerization.^29^ The actin regulates both synaptic structure and function.^15^ In this study, we first predetected the expression of Robo1 in the hippocampus of mice in a model of chronic pentatetrazin‐ignited epilepsy. It was found that Robo1 was basically expressed in the hippocampus of mice, suggesting that Robo1 is also involved in the pathogenesis of epilepsy. We observed that Robo1 was labeled by immunofluorescence. In the normal group, the protein expression of mice in the epileptic group was significantly increased. The hippocampus is an important part of epileptic occurrence. Robo1 was expressed on the membrane of hippocampal neurons of epileptic mice. For further confirmation, LV intervention was carried out, and the group with the most obvious knockout effect was selected from the three LV sequences for follow‐up experiments. The model of chronic PTZ‐lit mice was established after the elimination of Robo1, and the effect on the behavior of epileptic mice was studied. It was confirmed that after the reduction of Robo1, the seizure latency was significantly prolonged and the seizure class was reduced in the epileptic mice. It was hypothesized that Robo1 expression could promote the occurrence of epilepsy, while the results of immunoprotein blotting also suggested that protein expression was reduced after downregulation of Robo1 compared with the epileptic group, but no significant difference was available with the normal group of mice. Based on the above results, we speculated that Robo1 may be involved in the pathogenesis of epilepsy.

Dendritic spines are an important structure for transmitting information to excitatory neurons. Their morphology, quantity, and density have been significantly changed in a variety of nervous system diseases.^30^ The density of dendritic spines in epilepsy is still controversial in the study of the density of dendritic spines. Studies have found that in the model of hyacinic acid epilepsy, 7 days after the onset of epilepsy, the density of dendrite spines in the CA1 region decreased significantly compared with the control group.^31^ In the PTZ model, the density of dendritic spines in the dentate gyrus decreased significantly at 3 days and increased significantly at 7 days.^32^ In the chronic lithium chloride‐pilocarpine epilepsy model, the mean density of spines in the dentate gyrus of rats in the epileptic model increased, but the number of spines in good conditions decreased.^33^ It may be related to various factors such as animal model, time node, partition, and so on. It is now generally accepted that synaptic connections in the hippocampus are a projection of penetrating fibers, which start from the internal olfactory zone to the dentate gyrus and reach granule cell mossy fibers. Then, these pass lateral branches through the CA3 zone and end in the CA1 zone.^34^, ^35^ In our experiments, we found that the density of dendritic spines in the CA1 region of mice in the epileptic group was significantly increased at 30 days compared to the control group in the PTZ model. Although the dendritic spine density increased in the PTZ + LV‐Robo1‐shRNA group compared with the normal control group, it was still significantly lower than that of the epileptic group. Behavioral analysis showed that epileptic mice treated with lentiviral intervention with Robo1 had lower seizure ictal grades and a prolonged latency compared to the epileptic group. These findings suggest that Robo1 may regulate seizures by influencing dendritic spine density.

However, the morphology was mainly pseudopodia type and slender type, indicating that the morphology and density of dendritic spines in the CA1 region were affected by the long‐term excitatory stimulation of the central nervous system via PTZ. We found that in the Robo1‐mediated LV intervention group, the mature dendritic spines of mushroom, short and thick types were higher than those from the other two groups, while the filopodia type was less. These results suggest that downregulation of Robo1 negatively regulates the occurrence of epilepsy by reducing the density of dendritic spines and maintaining a greater number of mature dendritic spines.

## 5 CONCLUSIONS

Robo1 was expressed in mice's hippocampus and widely distributed in the membrane of neurons. LV‐mediated silencing of Robo1 prolonged seizure latency and reduced seizure grade. It is hypothesized that Robo1 may be involved in epileptogenesis by regulating the morphology and density of parietal dendritic spines in the CA1 region of the hippocampus.

## AUTHOR CONTRIBUTIONS

Hao Huang, Chang‐Yin Yu and Zu‐Cai Xu conceived and designed the study; Hao Huang, Zheng Liu and Wei Huang contributed to acquisition of data, analysis and interpretation; Man‐Min Zhu and Zhong‐Xiang Xu drafted the manuscript and assisted in the preparation of the figures. All authors have read and approved the final version of the manuscript.

## CONFLICT OF INTEREST STATEMENT

Zu‐Cai Xu is Associate Editor of Ibrain Journal editorial board. He is not involved in the peer review and editorial decision‐making processes of this article. The remaining authors declared they have no competing interests. [Correction added on 8 December 2023 after first online publication: This section was revised at the request of authors.]

## ETHICS STATEMENT

Animal experiments were conducted in accordance with the review and approval of the Animal Ethics Review Committee Office of Zunyi Medical University, Ethics Review number: KLLY (A)‐2020‐097.

## Supporting information

Supporting information.

## Data Availability

The data that support the findings of this study are available from the corresponding author upon reasonable request.
