# Supplementary material for: Mechanism of Robo1 in the pentylenetetrazol‐kindled epilepsy mouse model
Source: Ibrain. 2023 Aug 15;9(4):369–80. doi: 10.1002/ibra.12127 (PMC11045194; doi:10.1002/ibra.12127)
Supplement: Supplementary file 1 — Supporting information. [file IBRA-9-369-s001.docx]

**References**

[1]Malkki H. 2014. Epilepsy-burning questions and emerging therapies. Nature Reviews Neurology 10:243. doi: 10.1038/nrneurol.2014.63

[2]Anuradha Singh , Stephen Trevick . The Epidemiology of Global Epilepsy [J]. Neurol Clin. 2016,34(4):837-847.

[3]Brose K, Bland KS, Wang KH, et,al. Slit proteins bind Robo receptors and have an evolutionarily conserved role in repulsive axon guidance [J]. Cell. 1999,96(6):795-806.

[4]Cho JH, Kam JW, Cloutier JF.et,al. Slits and Robo-2 regulate the coalescence of subsets of olfactory sensory neuron axons within the ventral region of the olfactory bulb [J].Dev Biol. 2012,371(2):269-79.

[5]Zhang HY, Zheng LF, Yi XN, et,al. Slit1 promotes regenerative neurite outgrowth of adult dorsal root ganglion neurons in vitro via binding to the Robo receptor [J]. J Chem Neuroanat. 2010,39(4):256-61.

[6]Blockus H, Chedotal A. Slit-Robo signaling [J]. Development. 2016,143(17):3037-44.

[7]Koohini Z, Koohini Z, Teimourian S. Slit/Robo signaling pathway in cancer; a new stand point for cancer treatment. [J].Pathol Oncol Res. 2019,25(4):1285–1293.

[8]Magdalena Götz, Stefan H Stricker,Go with the flow: signaling from the ventricle directs neuroblast migration [J]. Nat Neurosci. 2006,9(4):470-2

[9]Kaneko N, Herranz-Pérez V, Otsuka T, et,al. New neurons use Slit-Robo signaling to migrate through the glial meshwork and approach a lesion for functional regeneration [J]. Sci Adv. 2018,4(12):eaav0618.

[10]Bing Chen, Lauren Carr, Xin-Peng Dun.Dynamic expression of Slit1-3 and Robo1-2 in the mouse peripheral nervous system after injury [J]. Neural Regen Res. 2020 May;15(5):948-958.

[11]Zhou Y,Luo Zhaohui,He Xinghui et al. Dynamic expression study of Robo3 in a rat model of temporal lobe epilepsy[J]. Journal of Epilepsy,2016,2(1):34-37.

[12]Barbara Schmeiser , Josef Zentner , Marco Prinz, et,al. Extent of mossy fiber sprouting in patients with mesiotemporal lobe epilepsy correlates with neuronal cell loss and granule cell dispersion [J].Epilepsy Res. 2017,129:51-58.

[13]Zheng W, Geng AQ, Li PF, et,al. Robo4 regulates the radial migration of newborn neurons in developing neocortex [J]. Cereb Cortex. 2012,22(11):2587-601.

[14]Guang Wang , Yan Li, Xiao-yu Wang, et,al. Slit/Robo1 signaling regulates neural tube development by balancing neuroepithelial cell proliferation and differentiation [J]. Exp Cell Res. 2013,319(8):1083-93.

[15]Nguemgo Kouam P, Rezniczek GA, Kochanneck A, et,al. Robo1 and vimentin regulate radiation-induced motility of human glioblastoma cells [J]. PLoS One. 2018,13(6):e0198508.

[16]Mariam C Recuenco , Tomoko Ohmori , Shunsuke Tanigawa , et,al. Nonmuscle Myosin II Regulates the Morphogenesis of Metanephric Mesenchyme-Derived Immature Nephrons [J]. J Am Soc Nephrol. 2015,26(5):1081-91.

[17]Kang Shen , Christopher W Cowan,et,al. Guidance Molecules in Synapse Formation and Plasticity [J]. Cold Spring Harb Perspect Biol. 2010,2(4):a001842

[18]SONG Zhaoying,GONG Jin,WANG Ying,et al. Plasticity changes in dendritic spines of hippocampal neurons in epileptic SD rats caused by sea manic acid[J]. Journal of Dalian Medical University,2019,41(5):389-395.

[19]Yu M. Effects of selective knockdown of hippocampal CA1 excitatory neurons Dnmt1 & Dnmt3a expression on memory and mechanism study[D]. 2018.

[20]Racine RJ. Modification of seizure activity by electrical stimulation. II.Motor seizure. Electroencephalogr Clin Neurophysiol. 1972;32:281–94.

[21]Huang H, Wang J, Zhang J, Luo Z, Li D, Qiu X, Peng Y, Xu Z, Xu P, Xu Z. Nitrobenzylthioinosine mimics adenosine to attenuate the epileptiform discharge of hippocampal neurons from epileptic rats. Oncotarget. 2017 May 30;8(22):35573-35582. doi: 10.18632/oncotarget.16012. PMID: 28415676; PMCID: PMC5482599.

[22]Robert S Fisher , Walter van Emde Boas, Warren Blume,et,al. Epileptic seizures and epilepsy: definitions proposed by the International League Against Epilepsy (ILAE) and the International Bureau for Epilepsy (IBE) [J]. Epilepsia. 2005,46(4):470-2.

[23]Chaithanya Reddy , Arushi Gahlot Saini , Metabolic Epilepsy[J].Indian J Pediatr. 2021 Oct;88(10):1025-1032.

[24]Luis A Martinez , Yi-Chen Lai , J Lloyd Holder Jr ,et,al. Genetics in Epilepsy[J]. Neurol Clin. 2021 Aug;39(3):743-777.

[25]Flavia Valtorta , Fabio Benfenati , Federico Zara et a1.PRRT2: from Paroxysmal Disorders to Regulation of Synaptic Function [J].Trends Neurosci. 2016 Oct;39(10):668-679.

[26]Yuan Jinxiao, Liu Xi, Ou Shu, et al. Study of spine morphology and functional remodeling in the formation of epilepsy[J]. Journal of the Third Military Medical University,2018,40(8):686-692.

[27]Yang Niu,Zhonghua Dai,Wenxue Liu,et a1.Ablation of SNX6 leads to defects in synaptic function of CA1 pyramidal neurons and spatial memory [J].Elife.2017 Jan 30;6:e20991.

[28]Ravasenga T,  Ruben M,  Regio V,  et,al. Spatial regulation of coordinated excitatory and inhibitory synaptic plasticity at dendritic synapses[J].Cell Rep 2022 02 08;38(6)

[29]WANG Xuefeng,J WANG Liang．Synaptic mechanism in pathogenesis of epilepsy[J].Journal of Chinese Neuroimmunology and Neurology,2010，17(4):235—237．

[30]KIM M, ROESENER AP, MENDONCA PR, et al. Robo1 and Robo2 have distinct roles in pioneer longitudinal axon guidance [J]. Dev Biol, 2011, 358(1): 181-188

[31]Wong K, Ren XR, Huang YZ, et,al.Signal transduction in neuronal migration: roles of GTPase activating proteins and the small GTPase Cdc42 in the Slit-Robo pathway[J].Cell 2001; 107(2): 209–221.

[32]CHIANG TS, LIN MC, TSAI MC, et al. ADP-ribosylation factor-like4A interacts with Robo1 to promote cell migration by regulating Cdc42 activation [J]. Mol Biol Cell, 2019, 30(1): 69-81

[33]ALEKSANDROVA N, GUTSCHE I, KANDIAH E, et al. Robo1 Forms a Compact Dimer-of-Dimers Assembly [J]. Structure, 2018, 26 (2): 320-328.e4.

[34]Gentile JE,  Carrizales MG,  Koleske AJ, Control of Synapse Structure and Function by Actin and Its Regulators[J].Cells 2022 Feb 09;11(4)

[35]NGUEMGO KOUAM P, REZNICZEK GA, KOCHANNECK A, et al. Robo1 and vimentin regulate radiation-induced motility of human glioblastoma cells [J]. PLoS One, 2018, 13(6): e0198508

[36]CHEN B, CARR L, DUN XP. Dynamic expression of Slit1-3 and Robo1-2 in the mouse peripheral nervous system after injury [J]. Neural Regen Res, 2020, 15(5): 948-958.

[37]PÉREZ C, SAWMILLER D, TAN J. The role of heparan sulfate deficiency in autistic phenotype: potential involvement of Slit/Robo/ srGAPs-mediated dendritic spine formation [J]. Neural Dev, 2016, 11: 11

[38]SHERCHAN P, HUANG L, AKYOL O, et al. Recombinant Slit2 reduces surgical brain injury induced blood brain barrier disruption via Robo4 dependent rac1 activation in a rodent model [J]. Sci Rep, 2017, 7(1): 746

[39]Khanal P,  Hotulainen P, Dendritic Spine Initiation in Brain Development, Learning and Diseases and Impact of BAR-Domain Proteins[J]. Cells 2021 09 12;10(9)

[40]Song Zhaoying, Gong Jin, Wang Ying, et al. Changes of plasticity of hippocampal neuronal dendritic spines in SD rats induced by sea human acid [J]. Journal of Dalian Medical University, 2018,41(5):389-395. (in Chinese)

[41]Xu Xiaoguang, Yu Shengbo, Gong Jin, et al. Germinating of granulosa cell dendrites in hippocampal dentate gyrus induced by pentatetrazide in epileptic rats [J]. Chin J Clinical Neurosurgery,2010(2).

[42]YUAN Jinxiao, LIU Xi, OU Shu, et al. Study of spine morphology and functional remodeling in the formation of epilepsy[J]. Journal of the Third Military Medical University,2018,40(8):686-692.

[43]Elisa Ren , Giulia Curia, Synaptic Reshaping and Neuronal Outcomes in the Temporal Lobe Epilepsy[J]. Int J Mol Sci. 2021 Apr 8;22(8):3860.

[44]Puhahn-Schmeiser B,  Leicht K,  Gessler F,et,al. Aberrant hippocampal mossy fibers in temporal lobe epilepsy target excitatory and inhibitory neurons[J].Epilepsia 2021 Oct;62(10)

.
